# A new through-the-scope clip with anchor prongs is safe and successful for a variety of endoscopic uses

**DOI:** 10.1055/a-2330-9803

**Published:** 2024-06-21

**Authors:** John J. Guardiola, Douglas K. Rex, Christopher C. Thompson, Jeffrey Mosko, Marvin Ryou, Joyce Peetermans, Matthew J Rousseau, Daniel von Renteln

**Affiliations:** 1 12250 Divsion of Gastroenterology and Hepatology, Indiana University School of Medicine, Indianapolis, United States; 2 1861 Division of Gastroenterology, Hepatology and Endoscopy, Brigham and Women's Hospital, Boston, United States; 3 10071 Gastroenterology, St Michael's Hospital, Toronto, Canada; 4 5724 Endoscopy, Boston Scientific Corp, Marlborough, United States; 5 5622 Gastroenterology, University of Montreal, Montreal, Canada

**Keywords:** Endoscopy Lower GI Tract, Endoscopic resection (polypectomy, ESD, EMRc, ...), Endoscopy Upper GI Tract, POEM, Gastrostomy and PEJ

## Abstract

**Background and study aims** Endoscopic through-the-scope clips (TTSC) are used for hemostasis and closure. We documented the performance of a new TTSC with anchor prongs.

**Patients and methods** We conducted a prospective case series of the new TTSC in 50 patients with an indication for endoscopic clipping at three hospitals in the United States and Canada. Patients were followed for 30 days after the index procedure. Outcomes included defect closure and rate of serious adverse events (SAEs) related to the device or procedure.

**Results** Fifty patients had 56 clipping procedures. Thirty-four procedures were clipping after endoscopic mucosal resection (EMR) in the colon (33) or stomach (1), 16 after polypectomy, two for hemostasis of active bleeding, and one each for fistula closure, per-oral endoscopic myotomy mucosal closure, or anchoring a feeding tube. Complete defect closure was achieved in 32 of 33 colon EMR defects and 21 of 22 other defects. All clips were placed per labeled directions for use. In 41 patients (82.0%), prophylaxis of delayed bleeding was reported as an indication for endoscopic clipping. There were three instances of delayed bleeding. There were no device-related SAEs. The only technical difficulty was one instance of premature clip deployment.

**Conclusions** A novel TTSC with anchor prongs showed success in a range of defect closures, an acceptable safety profile, and low incidence of technical difficulties.

## Introduction

Through-the-scope endoscopic clips (TTSCs) are used for a broad range of indications, including closure of gastrointestinal defects, with decreasing closure efficacy for larger defects 1 . Many types of TTSCs are available, and physicians often select clips based on physical characteristics, cost, or functional characteristics 2 . Defect characteristics and TTSC capabilities should be considered when choosing the closure device and technique 1 . Defect size is an important determinant of clip choice, i.e. small perforations that occur during endoscopic polyp resection can successfully be closed using TTSCs with favorable outcomes, while larger perforations are more expediently and effectively closed with large-caliber over-the-scope clips (OTSCs) mounted on the outside of a clear cap affixed to the tip of the endoscope 3 .

A 2019 bench study tested five models of marketed TTSCs with respect to rotatability, overshoot/whip, open/close precision, and tensile/closure strength in four different endoscope configurations: (1) straight, (2) duodenal sweep, (3) full retroflexion, and (4) across the duodenoscope elevator 2 . The authors mentioned that clip use in confined spaces or with thin tissue (e.g., Zenker’s diverticulectomy, endoscopic submucosal dissection-induced defects or bleeding) probably calls for a smaller clip with precise open/close, while therapies in full retroflexion or across the duodenoscope elevator probably require precise clip functioning in the most strained configuration 2 . Perforation closure may require clips with enhanced ability to laterally manipulate tissue to appose defect edges, and large ulcers or fibrous ulcers may require a clip with higher compression force 2 . Because increased gripping ability might expand potential indications, we are testing a TTSC with anchor prongs. Use of this clip has been reported for closure of complex polyp resection defects in three patients 4 . For preliminary evidence of the functional capacity of the new TTSC, we present an analysis of our first 50 cases.

## Patients and methods

### Study design

This was a case series of 50 consecutive patients with indication for endoscopic clipping, who received treatment with the MANTIS clip (Boston Scientific Corporation, Marlborough, Massachusetts, United States). This clip was cleared by the US Food and Drug Administration in August 2022 5 6 . The study was conducted at two US sites and one Canadian site.

### Patient population

Patients of any age scheduled for an indicated endoscopic clipping per local standard of practice were eligible to enroll. Exclusion criteria were enrollment in another study that would directly interfere with the current study or investigator’s assessment that the subject was at risk for study device or procedure-related complications. All centers obtained approval from their respective local ethics committees. All patients provided signed informed consent before nonemergent procedures. In emergent cases (e.g. perforation or acute bleeding), consent was performed after the procedure, but before data collection because preprocedural consent was unfeasible. Periprocedural management of antiplatelet and anticoagulant agents was per endoscopist discretion.

### Study visits

#### Index procedure and postprocedure follow-up

Baseline screening visit included informed consent, eligibility assessment, age, gender and relevant medical history. Intervention was placement of a new endoscopic clip in the gastrointestinal tract. During the index procedure, the type of clipping procedure, number of clips placed, and lesion size were recorded. After the index procedure, participants continued to receive medical care per standard of practice. Adverse events (AEs) and device events were followed for 30 days. The last study visit was a telephone interview at 30 days (± 5 days). For participants who did not complete this interview, the reason was recorded.

### Outcomes

We evaluated: 1) successful closure of defects defined as no submucosa visible and clips < 1 cm apart; 2) delayed bleeding rate; 3) reinterventions; 4) rate of serious AEs (SAEs) related to MANTIS clip or endoscopic portion of procedure; and 5) technical difficulties.

### Statistical analysis

Baseline characteristics, medical history, outcome measures, and AEs were summarized using mean, median, standard deviation (SD), range for continuous variables (e.g. age, procedure times), and proportions for categorical variables. All analyses were performed in SAS version 9.4.

## Results

### Patient and procedure characteristics

Among 63 patients screened for study eligibility, 10 were excluded for not having an indication for clip placement and three were excluded because the investigator deemed them at risk for study device or procedure-related complications per the Instructions for Use. Among the latter three patients, one defect was deemed too fibrotic, there was concern about placing both jaws in the center of a hot endoscopic mucosal resection (EMR) defect in the rectum of the second, and there was concern about placing both jaws of the MANTIS in submucosa to manage a vessel with ongoing bleeding in EMR defect in the third patient. Standard TTSCs were used in these cases. Among enrolled participants, the mean age was 62.8 years (range 28.0–80.0 years) and most participants were male (n = 32, 64.0%) and White (n = 44, 88.0%). Sixteen (32.0%) were taking nonsteroidal anti-inflammatory drugs including aspirin, six (12.0%) were taking anticoagulants, and two (4.0%) were taking antiplatelets at baseline ( Table 1 ).

**Table:** **Table 1** Baseline patient characteristics (n = 50).

Characteristic	Mean ± SD (range) or n/N (%)
**Mean age, yrs**	62.8±11.9 (28.0, 80.0)
**Gender: Male**	32/50 (64.0%)
**Ethnicity**	
Not Hispanic or Latino	45/50 (90.0%)
Hispanic or Latino	1/50 (2.0%)
Not disclosed	4/50 (8.0%)
**Race**	
White	44/50 (88.0%)
Black or African American	3/50 (6.0%)
American Indian or Alaska Native	1/50 (2.0%)
Hispanic or Latino	1/50 (2.0%)
Not disclosed	1/50 (2.0%)
Medical history*	
No medical history conditions	36/50 (72.0%)
Coronary artery disease	9/50 (18.0%)
Bleeding risk	5/50 (10.0%)
Chronic kidney disease	4/50 (8.0%)
Congestive heart failure	4/50 (8.0%)
Chronic obstructive pulmonary disease	1/50 (2.0%)
Esophageal dysphagia	1/50 (2.0%)
Known elevated bilirubin	1/50 (2.0%)
Liver disease	1/50 (2.0%)
Lung cancer	1/50 (2.0%)
Recurrent pneumonia	1/50 (2.0%)
**Actively taking NSAIDs**	16/50 (32.0%)
**Actively taking anticoagulants**	6/50 (12.0%)
Apixaban	3/50 (6.0%)
Lovenox	2/50 (4.0%)
Warfarin	1/50 (2.0%)
**Actively taking antiplatelets** (clopidogrel)	2/50 (4.0%)
POEM, peroral endoscopy myotomy. *Each patient had one or more of the listed medical conditions; rows are not mutually exclusive.	

### Types of procedures performed

Post-EMR prophylactic hemostasis was the reason for clipping in 34 defects. EMR was performed for 33 colonic lesions and one gastric lesion. Some patients had multiple lesions and thus underwent EMR of multiple lesions or a combination EMR/hot snare polypectomy. Sixteen colon polyps were removed from 12 patients via polypectomy without EMR – 15 using hot snare and one using cold snare. These resection defects were then clipped. Clipping was performed in two patients (4.0%) for hemostasis of active bleeding. Clipping was performed in one patient (2.0%) each for gastrocutaneous fistula closure 5 , post peroral endoscopic myotomy (POEM) closure of the mucosal incision, or anchoring of a feeding tube ( Table 2 ).

**Table:** **Table 2** Procedure details (N = 50).

Characteristic	Mean ± SD or median (range) or % (n/N patients)
**Reason for clipping procedure**	
Post-endoscopic mucosal resection (EMR)*	66.0% (33/50)
Polypectomy	24.0% (12/50)
Hemostasis of active bleeding	4.0% (2/50)
Fistula closure	2.0% (1/50)
Post-POEM mucosal incision closure	2.0% (1/50)
Anchoring/affixing of jejunal feeding tube	2.0% (1/50)
**Additional modalities used during procedure**	
None	86.0% (43/50)
Argon plasma coagulation with or without ligation ^†^	4.0% (2/50)
Endoscopic suturing - X-tack	4.0% (2/50)
Snare tip soft coagulation for active bleeding	4.0% (2/50)
Hemostatic grasper	2.0% (1/50)
Median maximum lesion diameter (range), mm	18.0 (1.2, 60.0)
Median minimum lesion diameter (range), mm	10.0 (1.0,40.0)
Mean number of study clips used per procedure (range)	2.6 ± 1.8 (1.0,9.0)
Mean number of non-study clips used per procedure (range)	1.0 ± 1.9 (0.0,9.0)
Mean total number of clips used per procedure (range)	3.6 ± 2.6 (1.0,11.0)
SD, standard deviation; EMR, endoscopic mucosal resection; POEM, peroral endoscopic myotomy; APC, argon plasma coagulation. *In the post-EMR category, one patient had three lesions, four patients had two lesions, and 28 patients had one lesion (total 39 lesions). Patients in all other categories had one lesion/clipping procedure each. ^†^ APC was used for margin and base ablation of a colonic EMR defect 6 7 and combined with ligature device for gastrocutaneous fistula closure 5 .	

### Study outcomes

#### Successful defect closure

Successful defect closure was completed in 53 of 55 lesions (96.4%) in 47 total patients in this study. The case in which endoscopic clipping was used for successful feeding tube anchoring is excluded here. Colonic post-EMR defects (33) were the most common lesions. All clips were placed per labeled directions for use. A post-EMR defect measuring 30 mm in maximum diameter x 5 mm in minimum diameter in the cecum was unable to be completely closed due to difficult positioning. A rectal polypectomy defect measuring 20 × 20 mm was incompletely closed due to inability to appose tissue.

Among the 55 defects, median maximum defect size was 18.0 mm (range 1.2–60.0 mm) and median minimum defect size was 10.0 mm (range 1.0–40.0 mm). The mean number of study clips used was 2.6 ± 1.8 (range 1.0–9.0) and the mean number of other (non-study) clips used was 1.0 ± 1.9 (range 0–9.0) per procedure. The mean total number of clips used during a procedure was 3.6 ± 2.6 (range 1.0–11.0).

For colon post-EMR defects, 32 of 33 defects were completely closed. The median minimum defect diameter was 10 mm (range 5–40 mm) and the median maximum defect diameter was 25 mm (range 8–45 mm) for these defects. For post-EMR defects of the colon, the mean number of clips placed was 4.4 ± 2.6 (range 1 to 11, including an average of 3.0 study clips and 1.3 non-study clips). Due to some defects lying over folds and other factors, the defect size was smaller than the actual lesion removed in some cases.

For non-colon EMR defects (excluding using a study clip to affix a jejunal tube), 21 of 22 were successfully completely closed. The median minimum diameter was 8 mm (range 1–40 mm) and the median maximum diameter was 10 mm (range 1.2–60 mm). For all non-EMR of colon defects, the mean number of clips placed was 2.7 ± 2.4 (range 1 to 11, including an average of 2.1 study clips and 0.6 non-study clips).

### Delayed bleeding prophylaxis

In 41 (82.0%) patient cases, prophylaxis to reduce risk of delayed bleeding was reported as the indication for endoscopic clipping. Delayed bleeding from clipped intervention sites occurred in three of 56 (5.4%) total interventions in 50 patients in the study. For post-EMR colon defects specifically, delayed bleeding occurred in one of 33 (3.0%) clipped defects. The one case of delayed bleeding was the previously mentioned incompletely closed cecal defect. For all other defects, delayed bleeding occurred in two of 22 clipped defects (9.0%). These cases were of the previously mentioned incompletely closed rectal polypectomy defect and the post-POEM defect.

### Serious adverse events related to the MANTIS clip

Of the 50 enrolled patients, none of the patients had AEs or SAEs related to the MANTIS clip nor any other clip.

### Study clip technical success

There was one instance of the clip deploying prematurely. There were zero instances of the clip failing to deploy, failing to release from the catheter, being difficult to release from the catheter, failing to open or close, being unable to rotate, failing to advance from the sheath, being difficult to deploy, having the clip arms or anchor prongs bent, having poor bite of tissue, or any other events that could be seen as technical failure of the clip itself.

### Serious adverse events related to the endoscopic procedure and reinterventions

Five patients had a total of four SAEs and one nonserious AE related to the endoscopic portion of the procedure within a median of 12 days (range 0–14 days) after the index procedure ( Table 3 ). The first patient had a POEM procedure with a related SAE and two reinterventions. The patient was found to have esophageal leak on imaging on Day 0 but the site was contained so the patient was treated with antibiotics and nothing by mouth. This patient had two repeat upper endoscopies. The patient had bleeding from the esophageal mucosectomy site and an esophageal ulcer. The first repeat endoscopy performed for bleeding on Day 13 after the procedure showed a clot near the clipping site; the study clip was removed for better visualization and the site was treated with a hemostatic spray and placement of a non-study clip. Two days later, this patient had another procedure to treat an esophageal ulceration using hemostatic spray. No blood transfusion was required. The second patient had a related SAE and reintervention due to bleeding on Day 12 after EMR of a cecal lesion that was incompletely closed due to difficult access. This patient underwent colonoscopy. The endoscopist surveilled the affected site, but no treatment was needed. No blood transfusion was required. A third patient who had a rectal hot snare polypectomy site that was not completely closed also had a bleed on post-procedure Day 6 that resolved (nonserious AE with a reintervention). Subsequently, colonoscopy was performed and no intervention was needed. No blood transfusion was needed. Notably, a fourth patient had an unrelated SAE with a reintervention after melena occurred on Day 1 after the procedure. An upper endoscopy demonstrated bleeding not related to the previous endoscopic procedure nor the study clip. This bleeding was treated with a non-study clip. A fifth patient had a related SAE of post-polypectomy electrocoagulation syndrome that did not require reintervention.

**Table:** **Table 3** Serious adverse events related to the endoscopic portion of the procedure.

	Number of SAEs	Number of patients (n/N) (%)
**Any serious adverse event**	5	3/50 (6.0%)
Bleeding	2	2/50 (4.0%)
Esophageal perforation	1	1/50 (2.0%)
Post-polypectomy electrocoagulation syndrome	1	1/50 (2.0%)
Ulceration	1	1/50 (2.0%)
SAE, serious adverse event.		

### Descriptions of how the clip was used

#### “Grasp-and-drag” technique for a standard EMR defect

**Part 1** “Grasp-and-drag” technique to close a defect after hot snare piecemeal endoscopic mucosal resection of a lateral spreading tumor in the colon. **Part 2** “Open-jaw” technique to prophylactically close a scarred defect of a hot endoscopic mucosal resection of a 50-mm scarred granular lateral spreading tumor in the ascending colon. **Part 3** Repeated “open-jaw” technique to prophylactically close a broad-based defect after piecemeal endoscopic mucosal resection of a 45-mm granular lateral spreading tumor in the cecum.
Video 1

After hot snare piecemeal EMR of a lateral spreading lesion in the colon, several non-MANTIS clips had already been placed. Video 1 demonstrates the MANTIS clip was opened and normal mucosa on one edge of the lesion was grasped. With the MANTIS clip closed, the colonoscope was used to lift the MANTIS clip with grasped tissue to the opposite side of the lesion. The MANTIS clip was then opened, with the anchor prongs maintaining the grasp of tissue. With the jaws open, the MANTIS clip was gently pushed to the mucosa to maximize use of its jaws, then closed approximating defect edges, then deployed ( Video 1 – Part 1).

#### “Open-jaw” technique for a scarred defect

The lesion was a 50-mm granular lateral spreading tumor (G-LST) in the ascending colon that had undergone a previous attempt at resection before referral to our center. The lesion was assessed and resected with piecemeal hot EMR. Evidence of the previous resection attempt was seen at the haustral folds as scarred-down tissue extending across the defect. Hot avulsion was used to remove flat and fibrotic polyp tissue. Snare tip soft coagulation was performed to the edges of the defect.

The MANTIS clip's anchor prongs were used to drag tissue to approximate the defect without closing and reopening the jaws (“open-jaw technique”) or slipping from the tissue as standard TTSCs might when used to close a wide defect. This technique is useful when the defect is approached tangentially. Here, the MANTIS clip was rotated so that when opened, the orientation was vertical with the top jaw between 10 and 2 o'clock. The bottom anchor prongs were used to grasp normal tissue at the distal (anal side) edge of the lesion and the colonoscope was advanced to lift and push the mucosa on the anal side of the defect toward the mucosal edge on the defect’s cecal side. The top anchor prongs were then used to grasp normal tissue at the proximal edge of the lesion. The clip was pushed gently to "bury" the clip and use the full length of the jaws before deployment. This sequence was repeated. The defect was closed with a total of six MANTIS clips and one standard TTSC. The MANTIS clip was easily maneuvered for placement between previously deployed clips. Alternatively, fewer MANTIS clips can be used to bring the defect edges closer, followed by closure of the remaining defect using standard TTSC ( Video 1 – Part 2).

#### “Open-jaw” technique for a broad-based defect

This shows a large submucosal defect after piecemeal hot EMR of a 45-mm G-LST in the cecum. The "open-jaw” technique was again utilized to grasp normal tissue on the anal side of the lesion and move it to the cecal edge of lesion. Three MANTIS clips were used followed by three standard TTSC to completely close this lesion ( Video 1 – Part 3).

## Discussion

A new TTSC with anchor prongs had a high rate of success for defect closure, low rate of delayed bleeds, and high rate of technical success regarding clip deployment in this case series of 50 patients across three centers in the United States and Canada in a variety of cases. There were zero AEs related to the clip itself and it was technically successful in all but one instance because of premature deployment. The low rate of related SAEs was consistent with published safety data on endoscopic clips 8 . We demonstrated two techniques for successful use of this technique.

Due to the tissue apposition ability of the MANTIS clip, it may be able to reduce the number of standard TTSCs used during defect closure or allow for closure of larger defects without OTSC or suturing devices. Recently, another novel TTSC has been introduced – the dual action tissue (DAT) clip (Micro-Tech Endoscopy, USA, Ann Arbor, Michigan, United States) 9 . This clip features two independent arms and is efficacious in closing large resection defects. Because it cannot be rotated, it requires manipulation of the scope dials for positioning and operating the independent jaws can be difficult with endoscope looping 10 . The MANTIS is operated similarly to a standard TTSC. It may have a shorter learning curve for both endoscopist and technician than alternative devices. Comparative studies for endoscopic defect closure between these clips and other devices such as suturing are warranted.

### Data sharing

The data, analytic methods, and study materials for this study may be made available to other researchers in accordance with the Boston Scientific Data Sharing Policy ( http://www.bostonscientific.com/en-US/data-sharing-requests.html ).
